# Cyclocreatine treatment ameliorates the cognitive, autistic and epileptic phenotype in a mouse model of Creatine Transporter Deficiency

**DOI:** 10.1038/s41598-020-75436-4

**Published:** 2020-10-27

**Authors:** Francesco Cacciante, Mariangela Gennaro, Giulia Sagona, Raffaele Mazziotti, Leonardo Lupori, Elisa Cerri, Elena Putignano, Mark Butt, Minh-Ha T. Do, John C. McKew, Maria Grazia Alessandrì, Roberta Battini, Giovanni Cioni, Tommaso Pizzorusso, Laura Baroncelli

**Affiliations:** 1 Institute of Neuroscience, National Research Council (CNR), Via Moruzzi 1, 56124 Pisa, Italy; 2 BIO@SNS Lab, Scuola Normale Superiore di Pisa, 56125 Pisa, Italy; 3 Department of Neuroscience, Psychology, Drug Research and Child Health NEUROFARBA, University of Florence, 50135 Florence, Italy; 4 Department of Developmental Neuroscience, IRCCS Stella Maris Foundation, 56128 Pisa, Italy; 5 Tox Path Specialists, Frederick, MD 21701 USA; 6 Lumos Pharma, Austin, TX 78756 USA; 7 Department of Clinical and Experimental Medicine, University of Pisa, 56126 Pisa, Italy

**Keywords:** Neuroscience, Diseases of the nervous system

## Abstract

Creatine Transporter Deficiency (CTD) is an inborn error of metabolism presenting with intellectual disability, behavioral disturbances and epilepsy. There is currently no cure for this disorder. Here, we employed novel biomarkers for monitoring brain function, together with well-established behavioral readouts for CTD mice, to longitudinally study the therapeutic efficacy of cyclocreatine (cCr) at the preclinical level. Our results show that cCr treatment is able to partially correct hemodynamic responses and EEG abnormalities, improve cognitive deficits, revert autistic-like behaviors and protect against seizures. This study provides encouraging data to support the potential therapeutic benefit of cyclocreatine or other chemically modified lipophilic analogs of Cr.

## Introduction

Creatine Transporter Deficiency (CTD) is an inborn error of metabolism with X-linked inheritance pattern (OMIM #300352). The clinical presentation of CTD includes brain creatine (Cr) shortage, mild to severe intellectual disability, behavioral disturbances or autistic-like features and epilepsy^1^, and typically arises between birth and 6 years of age^2^.

Although rare, CTD represents a major issue in health-care, causing chronic illness with a considerable impact on the everyday quality of life of patients and caregivers. Previous attempts to rescue creatine (Cr) content in the brain and attenuate the symptoms of CTD children by exploiting nutritional supplements based on Cr alone and/or administered together with its synthesis precursors have been of very limited success^3–8^. Thus, there are currently no satisfactory treatments for this disorder, and the mainstay of care is a palliative approach for managing seizures and behavioral problems. In recent times, knock-out murine models for the creatine transporter (CrT) gene showing high face validity and the discovery of novel quantitative biomarkers have led to a significant leap for the development of potential therapeutic agents^9–15^.

Here, we performed a randomized, blinded, placebo-controlled, preclinical study to explore the therapeutic efficacy of a chronic (24 week) oral daily treatment with cyclocreatine (cCr) at three different dose levels in CrT knock-out (CrT^−/y^) mice. It has been reported that lipophilic analogs and other derivatives of Cr can enter cells independently of CrT and could represent a promising approach for CTD treatment^10,16–19^. In particular, cCr is a nearly planar Cr analog that can be phospho/dephosphorylated by Cr kinase, thus mimicking the metabolic function of Cr^10,20,21^. However, the evaluation of cCr and other treatment options in CTD has been limited to biochemical measurements, behavioral outcomes and in vitro assays of neuronal function. We combined functional biomarker assessment with well-established behavioral readouts for CTD^11,15^ to monitor brain function of CrT^−/y^ mice in response to pharmacological intervention with cCr.

## Results

**Figure 1:**
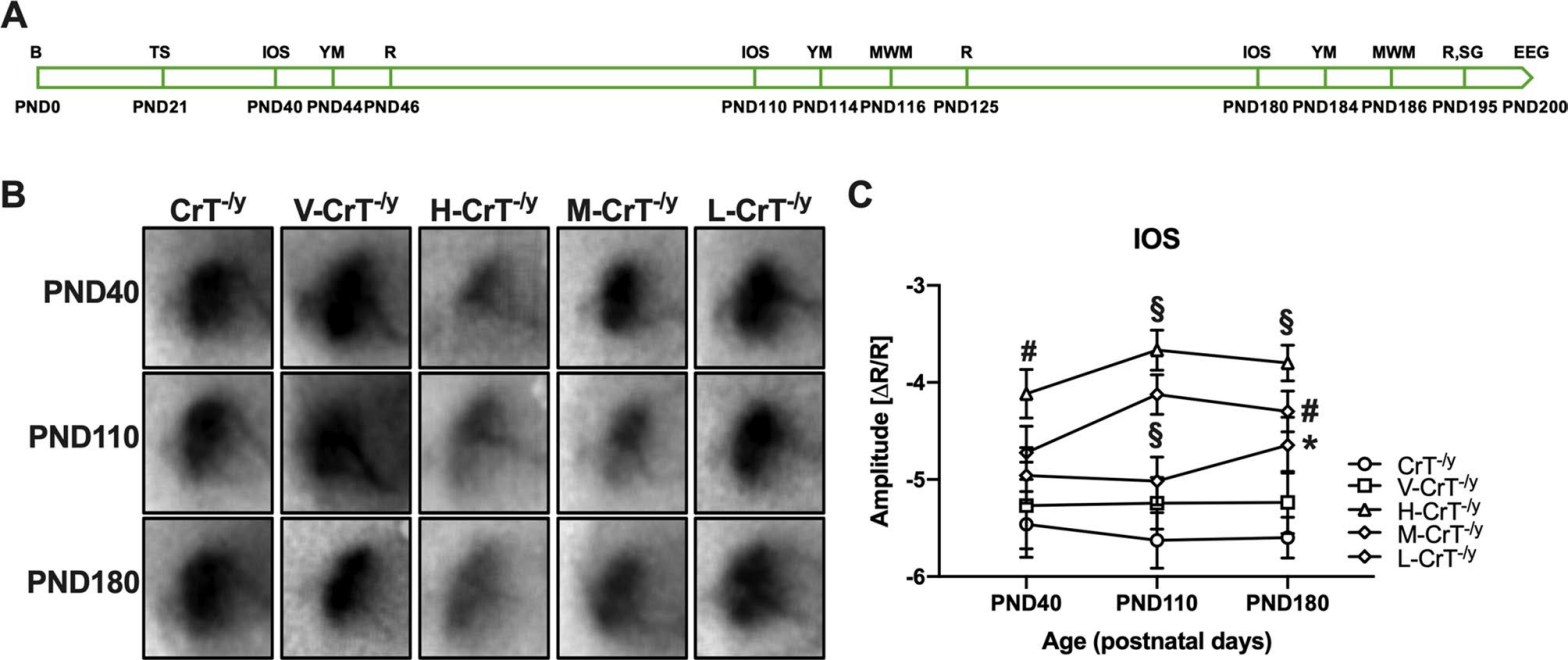
cCr treatment improves hemodynamic responses in CrT^−/y^ mice. (**A**) Schematic diagram of the experimental design. We employed IOS imaging, behavioral assessment (Y maze, Morris water maze, rotarod and self-grooming) and video-EEG recordings to evaluate the therapeutic efficacy of longitudinal treatment with cCr at three different doses (high dose, H: 140 mg/kg, medium dose, M: 46 mg/kg, low dose, L: 14 mg/kg). Untreated mice (CrT^−/y^) and animals administered with placebo (only chocolate milk, V-CrT^−/y^) were used as controls. List of abbreviations—B: birth; TS: treatment starts; IOS: intrinsic optical signal; YM: Y maze; R: rotarod; MWM: Morris Water Maze; SG: self-grooming; PND: postnatal day. (**B**) Representative IOS response images collected at different ages (PND40, PND110 and PND180) in CrT^−/y^, V-CrT^−/y^, H-CrT^−/y^, M-CrT^−/y^ and L-CrT^−/y^ mice (n = 15 for each group at each time point, except n = 17 each for CrT^−/y^, H-CrT^−/y^ and M-CrT^−/y^ mice at PND180). (**C**) Mean amplitude of visually evoked IOS responses measured after 20 (PND40), 90 (PND110) and 160 (PND180) days of cCr delivery. Two-way ANOVA revealed a significant effect of treatment (*p* < 0.001) and post-hoc Dunnett’s multiple comparisons test showed an amelioration of hemodynamic responses in H-CrT^−/y^ mice at every time point tested (*p* < 0.01 at PND40, *p* < 0.001 at PND110 and PND180), whereas IOS signals were attenuated at PND110 (*p* < 0.001) and PND180 (*p* < 0.01) in M-CrT^−/y^ animals, and only at PND180 (*p* < 0.05) in L-CrT^−/y^ mice. No difference was present between untreated CrT^−/y^ animals and V-CrT^−/y^ mice (*p* = 0.965 at PND40, *p* = 0.728 at PND110, *p* = 0.739 at PND180). * *p* < 0.05, # *p* < 0.01, § *p* < 0.001. Error bars, SEM.

cCr was administered orally once daily at three different doses (high dose, H: 140 mg/kg, medium dose, M: 46 mg/kg, low dose, L: 14 mg/kg) to CrT^−/y^ mice and wild-type littermates starting at postnatal day (PND) 21. We longitudinally evaluated the therapeutic efficacy of cCr treatment in the same animals, using intrinsic optical signal (IOS) imaging, a panel of behavioral test (Y maze, Morris water maze, rotarod, self-grooming) and video-EEG recordings (Fig. 1a).

### cCr treatment improves cortical hemodynamics in CrT^−/y^ mice

Since we recently reported that hemodynamic responses are altered in the visual cortex CrT^−/y^ mice with no impairment of visually-evoked potentials and that hemodynamic measurements are a reliable biomarker of brain function in CTD^15^, we first explored the therapeutic efficacy of cyclocreatine (cCr) using IOS imaging (Fig. 1a). We found a dose- and age-dependent effect on hemodynamic responses in CrT^−/y^ mice administered cCr. The altered IOS amplitude observed in CrT^−/y^ mice was significantly attenuated in CrT^−/y^ mice treated with the high dose of cCr (H-CrT^−/y^) at every time point tested. The medium dose (M-CrT^−/y^) ameliorated IOS signals at PND110 and PND180, and the low dose (L-CrT^−/y^) was effective only at PND180 (Fig. 1b,c, Fig. S1). No difference was detected between untreated animals (CrT^−/y^) and vehicle-treated mice (V-CrT^−/y^, Fig. 1b,c, Fig. S1). These data suggest that cCr delivery is able to circumvent, at least partially, the metabolic impairment caused by Cr depletion, leading to the reversion of the neurovascular phenotype characterizing CrT^−/y^ mice.

### cCr reduces deterioration of cognitive functions in CrT^−/y^ mice

**Figure 2:**
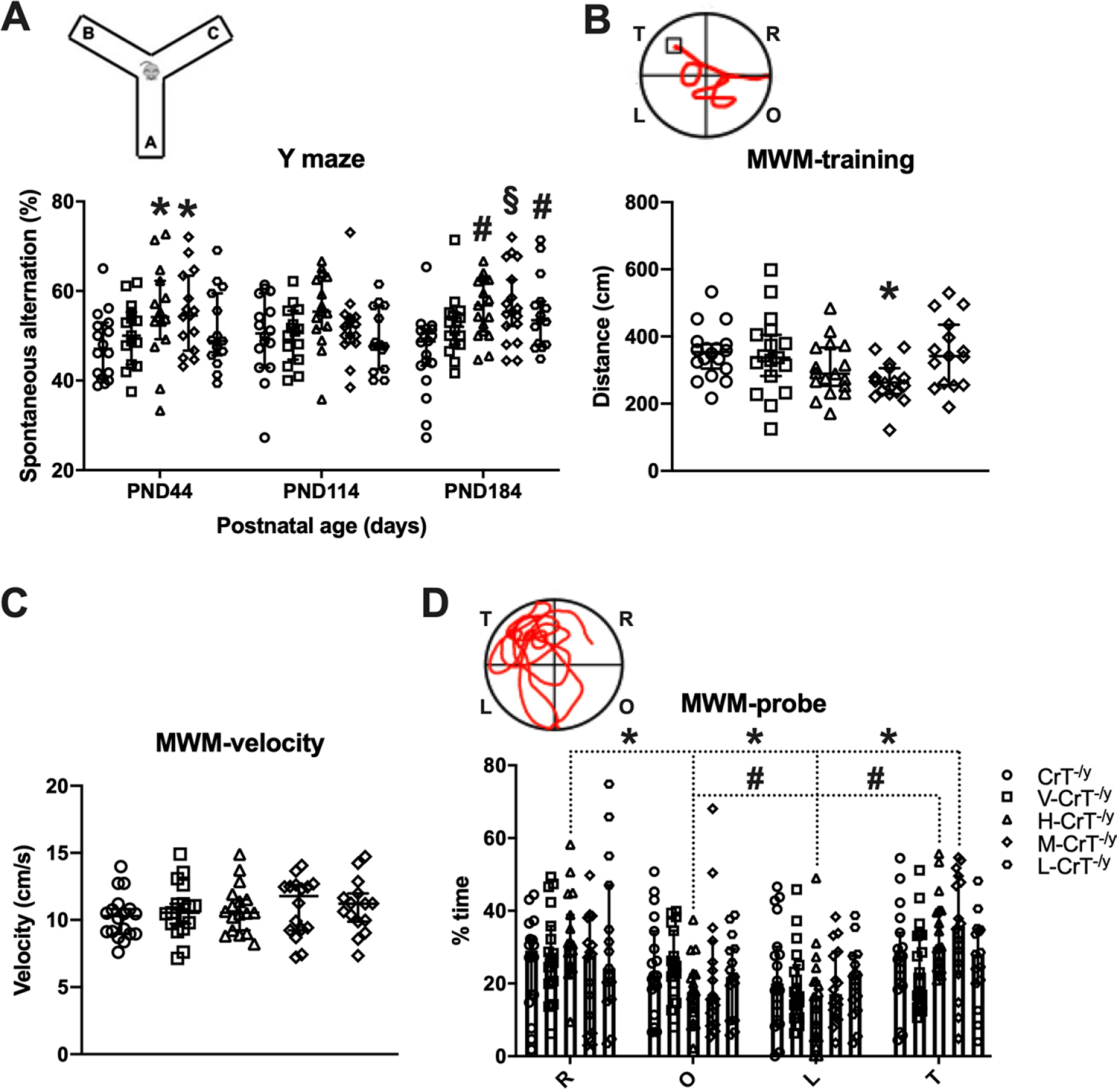
cCr treatment attenuates cognitive deterioration in CrT^−/y^ mice. (**A**) Histograms depict the spontaneous alteration rate in the Y maze for the different experimental groups at PND44, PND114 and PND184 (n = 15 for each group at each time point, except n = 17 each for CrT^−/y^, H-CrT^−/y^ and M-CrT^−/y^ mice at PND180). The performance of H-CrT^−/y^ and M-CrT^−/y^ animals was statistically higher than CrT^−/y^ littermates at PND44 (Two-way ANOVA, effect of treatment *p* < 0.001, post-hoc Dunnett’s multiple comparisons test, *p* < 0.05) and PND184 (*p* < 0.01 for H-CrT^−/y^ group, *p* < 0.001 for M-CrT^−/y^ mice); L-CrT^−/y^ showed a slightly improved spontaneous alternation only at PND184 (*p* < 0.01). No difference emerged between CrT^−/y^ animals and V-CrT^−/y^ mice at every age tested (*p* = 0.767 at PND44, *p* = 0.999 at PND114, *p* = 0.112 at PND184). The inset depicts a schematic diagram of the Y maze apparatus. (**B**–**D**) MWM at PND186 (n = 15 for each group, except n = 17 each for CrT^−/y^, H-CrT^−/y^ and M-CrT^−/y^ mice). The mean swimming path covered prior to locate the submerged platform on the last three days of training (**B**) was statistically shorter in M-CrT^−/y^ mice (One-way ANOVA, p < 0.05, post-hoc Dunnett’s multiple comparisons test, *p* < 0.05). The inset shows a representative example of swimming path during the training phase. Mean swimming speed (**C**) was unaffected by cCr treatment (One-way ANOVA, *p* = 0.774). Open symbols represent single data values; black lines indicate median with 95% CI. (**D**) Probe trial. A Two-Way RM ANOVA followed by Dunnett’s multiple comparisons revealed that while CrT^−/y^ animals did not show any exploration preference, H-CrT^−/y^ mice spent significantly more time in the T quadrant than in the opposite (O, *p* < 0.01) and in the left (L, *p* < 0.01) quadrants. No difference was present between the time in the T and the right (R) quadrant (*p* = 0.968). Moreover, a significant difference was detected between the time in R quadrant and O (*p* < 0.05) and L (*p* < 0.05) quadrants. M-CrT^−/y^ mice spent significantly more time in the T quadrant than in the L quadrant (*p* < 0.01). No effect of the low cCr dose or vehicle treatment was detected. The inset shows a representative example of swimming path during the probe phase. * *p* < 0.05, # *p* < 0.01, § *p* < 0.001. Open symbols represent single data values; histograms and lines indicate median with 95% CI.

After each IOS session, mice were subjected to serial neurobehavioral assessments of cognitive and psychomotor functions (Fig. 1a). We previously reported that CrT^−/y^ animals exhibit a general cognitive impairment across different learning and memory tasks^11^. The performance in the Y maze revealed a moderate but statistically significant increase in the spontaneous alternation for H-CrT^−/y^ and M-CrT^−/y^ mice at PND44, indicating a cognitive improvement in these groups of animals. No beneficial effects of treatment were detected at PND114, while all three cCr doses comparably improved working memory at PND184 (Fig. 2a). V-CrT^−/y^ showed an alternation rate comparable to untreated animals (Fig. 2a). We further assessed memory abilities using the Morris Water Maze (MWM) at PND116 and PND186. While no positive effects of cCr administration were detected at PND116 (Fig. S2), M-CrT^−/y^ mice were significantly stronger learners compared to age-matched untreated CrT^−/y^ animals at PND186, with a shorter distance to locate the platform during the training phase (Fig. 2b). The lack of impact of cCr treatment on swimming velocity (Fig. 2c) attributes this improvement in distance to learning, rather than to any impact of cCr on skeletal muscle. Although there was no unilateral preference for the target quadrant in the probe trial, H-CrT^−/y^ and M-CrT^−/y^ animals mostly explored the target quadrant and the adjacent one, suggesting the presence of a memory trace (Fig. 2d). No difference was observed between untreated and vehicle-treated CrT^−/y^ animals (Fig. 2b–d). Altogether, these results suggest that cCr has a partial effect in restoring the learning and memory functions deteriorated in CrT deficiency conditions. On the other hand, cCr treatment did not result in any change of body weight (Fig. S3), substantiating the lack of impact of cCr treatment on skeletal muscle observed in the MWM.

### Repetitive and stereotyped behavior of CrT^−/y^ mice is reverted following cCr treatment

**Figure 3:**
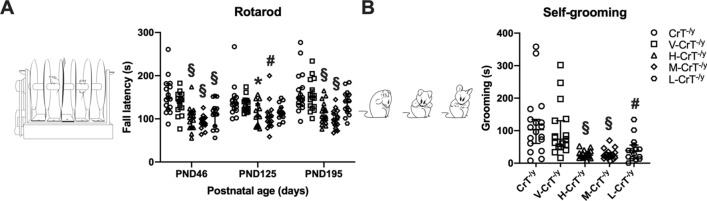
cCr treatment robustly decreases stereotyped behaviors in CrT^−/y^ mice. (**A**) A Two-way ANOVA highlighted a significant effect of treatment (*p* < 0.001) in the performance of CrT^−/y^ animals on the accelerating rotarod (n = 15 for each group at each time point, except n = 17 each for CrT^−/y^, H-CrT^−/y^ and M-CrT^−/y^ mice at PND180). Post hoc Dunnett’s multiple comparison test showed that the fall latency of H-CrT^−/y^, M-CrT^−/y^ and L-CrT^−/y^ mice was significantly lower from that of CrT^−/y^ animals at PND46 (*p* < 0.001 for all groups), whereas only H-CrT^−/y^ and M-CrT^−/y^ mice exhibited an improved performance at PND125 (*p* < 0.05 for H-CrT^−/y^ and *p* < 0.01 for M-CrT^−/y^) and PND195 (*p* < 0.001 for both groups). No difference was present between CrT^−/y^ animals and V-CrT^−/y^ mice (*p* = 0.293 at PND46, *p* = 0.876 at PND125, *p* = 0.888 at PND195). Inset shows an illustration of the rotarod apparatus. (**B**) H-CrT^−/y^, M-CrT^−/y^ and L-CrT^−/y^ mice showed decreased self-grooming behavior at PND195 (n = 15 for each group, except n = 17 each for CrT^−/y^, H-CrT^−/y^ and M-CrT^−/y^ mice; One-way ANOVA, effect of treatment *p* < 0.001, *post*-hoc Dunnett’s multiple comparisons test, *p* < 0.001 for H-CrT^−/y^ and M-CrT^−/y^ animals, *p* < 0.01 for L-CrT^−/y^ mice). No effect of vehicle treatment was detected (*p* = 0.793). A schematic representation of self-grooming behavior is also shown. * *p* < 0.05, # *p* < 0.01, § *p* < 0.001. For all panels, open symbols represent single data values; black lines indicate median with 95% CI.

We formerly outlined that, despite the lack of differences in sociability, stereotyped behaviors in the rotarod and self-grooming test seem to be amplified in CrT^−/y^ mice, demonstrating that the endophenotype of this mouse model includes autistic-like features^11,22–29^. Here, we found that all three dose levels of cCr were effective in reducing the manifestation of stereotyped movements in the rotarod task at PND46, while at PND125 and PND195 only H-CrT^−/y^ and M-CrT^−/y^ animals displayed the same beneficial effect (Fig. 3a). These results were also corroborated by the measurement of self-grooming, with H-CrT^−/y^, M-CrT^−/y^ and L-CrT^−/y^ mice showing a drastic reduction in the time spent grooming themselves at PND195 (Fig. 3b). Thus, these data further support the beneficial activity of cCr at the level of the brain and suggest that cCr might have the ability to improve ASD-related symptoms in CTD patients.

### cCr protects against spontaneous epileptic phenotype and increases resistance to chemically-induced seizures

**Figure 4:**
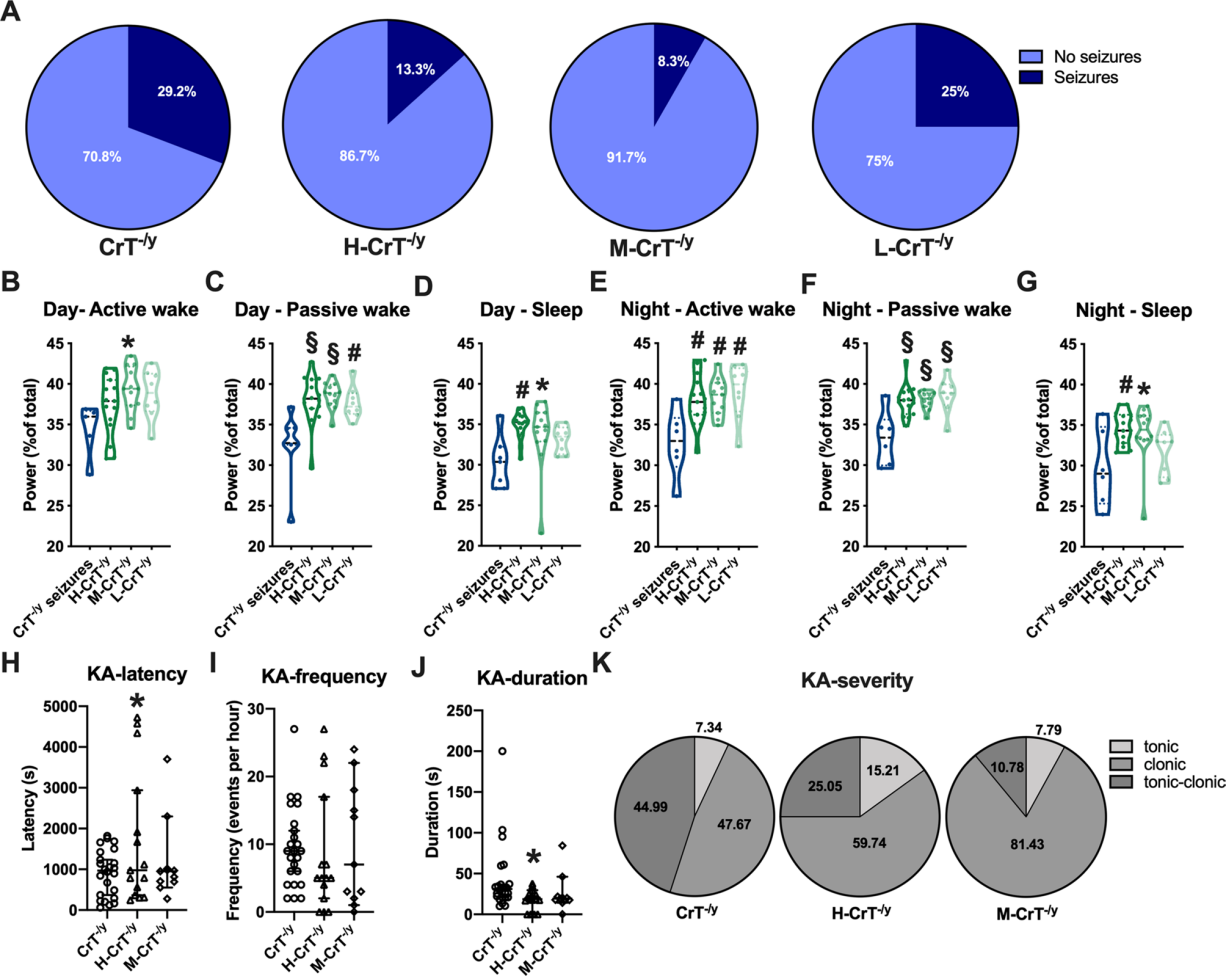
cCr treatment protects CrT^−/y^ mice against spontaneous and evoked epilepsy. (**A**) The percentage of animals experiencing spontaneous seizures at the clinical and electrographical level was significantly lower in H-CrT^−/y^ (n = 15; Fisher’s exact test, *p* < 0.01) and M-CrT^−/y^ mice (n = 12; *p* < 0.001) than in CrT^−/y^ group (n = 24). No beneficial effect was detected in L-CrT^−/y^ animals (n = 9; *p* = 0.431). Untreated and V-CrT^−/y^ mice were pooled together in the CrT^−/y^ group. (**B**–**G**) The comparison of power spectra of cortical EEG recordings with epileptic CrT^−/y^ mice (CrT^−/y^ -seizures, n = 7) revealed an increased power of 4–8 Hz theta frequency in cCr-treated animals (One-way ANOVA, post-hoc Dunnett’s multiple comparisons test). Violin plots show individual values and the entire distribution of data. (**H**–**K**) Effect of KA treatment in CrT^−/y^, H-CrT^−/y^ and M-CrT^-/^ mice at behavioral and electrophysiological level. While no difference was present in the frequency of ictal events (**I**, One-way ANOVA, *p* = 0.897), H-CrT^−/y^ mice displayed a longer latency (**H**, One-way ANOVA, effect of treatment *p* < 0.05, post-hoc Dunnett’s multiple comparisons test, *p* < 0.05) and a shorter duration (**J**, One-way ANOVA, effect of treatment *p* < 0.05, post-hoc Dunnett’s multiple comparisons test, *p* < 0.05) of epileptiform bursts compared to CrT^−/y^ animals. Open symbols represent single data values; black lines indicate median with 95% CI. (**K**) Relative percentage of tonic, clonic and tonic–clonic seizures indicates that seizure severity is less pronounced in H-CrT^−/y^ and M-CrT^−/y^ animals at 10 mg/kg (χ^2^ test; *p* < 0.001). * *p* < 0.05, # *p* < 0.01, § *p* < 0.001.

To assess the effects of cCr treatment on the epileptic phenotype recently observed in CrT^−/y^ mice^15^, we used video-EEG recordings in freely-moving animals. The percentage of subjects experiencing spontaneous seizures at the clinical and electrographical level was significantly lower in H-CrT^−/y^ and M-CrT^−/y^ groups (Fig. 4a) and a significant increase of theta band power was detected in the brain of CrT^−/y^ mice treated with cCr compared to untreated CrT^−/y^ animals showing spontaneous seizures (Fig. 4b–g; Fig. S4). Spectral analysis also revealed a higher alpha frequency in H-CrT^−/y^ mice during sleep (Fig. S4).

Based on these results, we assessed the response to KA challenge in H-CrT^−/y^ and M-CrT^−/y^. Electrographical analysis and behavioral scoring revealed that H-CrT^−/y^ animals display a more prolonged latency and a shorter duration of epileptiform bursts (Fig. 4h,j), although cCr treatment did not affect the frequency of evoked seizures (Fig. 4i). The distribution of seizure severity was also statistically different among the different groups, with H-CrT^−/y^ and M-CrT^−/y^ mice presenting a lower number of tonic–clonic events (Fig. 4k).

Taken together, these results suggest that cCr can protect against spontaneous seizures and shift epileptic susceptibility towards a milder phenotype.

### cCr permeates the blood–brain barrier and gets into the brain

**Figure 5:**
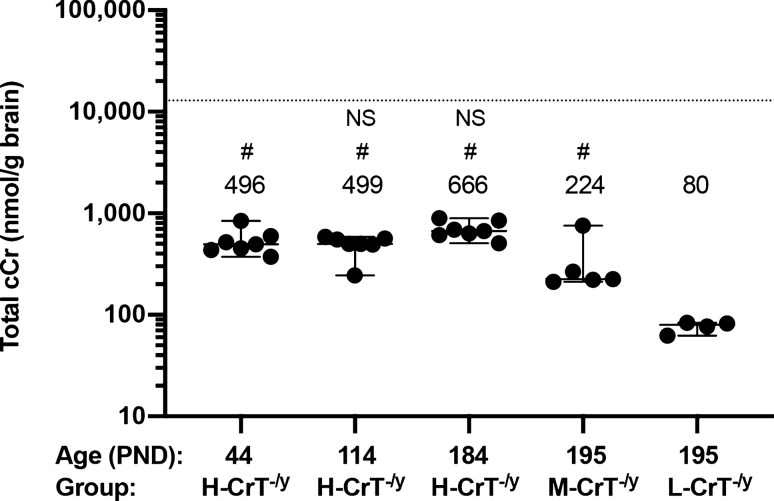
Total brain cCr following cCr treatment. Concentrations of total cCr (summation of cCr and phospho-cCr) were measured in satellite H-CrT^−/y^ mice at PND44, PND114 and PND184 (n = 7 for each time point), and in a subset of M-CrT^−/y^ (n = 5) and L-CrT^−/y^ (n = 4) mice from the efficacy study at PND195 by LC–MS/MS. Circles represent single data values; black lines indicate medians with 95% CI. The dashed line represents median total creatine (creatine plus phospho-creatine) measured in brains of wild-type male C57BL/6J mice at PND28-35. One-way ANOVA, post-hoc Dunnett’s multiple comparisons test: # *p* ≤ 0.05 from L-CrT^−/y^, NS not statistically different from H-CrT^−/y^ at PND 44.

To assess the brain penetration of cCr, we monitored the pharmacokinetic profile in a group of animals following oral administration. We detected brain entry of cCr in all mice chronically treated with the drug, with higher levels of total cCr in H-CrT^−/y^ mice than in M-CrT^−/y^ and L-CrT^−/y^ animals (Fig. 5). Moreover, longitudinal quantification of cCr at the three different ages employed for behavioral and functional testing revealed that high-dose administration promptly leads to a rise in cCr concentration in the brain, with cCr reaching steady state levels following 23 days of dosing and remaining stable through the treatment period (Fig. 5).

### No pathological changes are associated with cCr dosing

No cCr-related deaths or overt signs of toxicity were observed at any dose. We also investigated whether cCr dosing was accompanied by pathological changes in brain morphology. Amygdaloid body, basal nuclei/striatum, cerebellum, cerebral cortex, hippocampus, hypothalamus, medulla oblongata, meninges, midbrain, olfactory bulb, pons, thalamus ventricular system, and white matter were evaluated in H-CrT^−/y^ mice using a standard Hematoxylin and Eosin (H&E) staining. No variations in the neuroanatomical architecture were observed (Fig. S5).

## Discussion

By using a wide battery of functional and behavioral assessment previously validated in this mouse model of CTD^11,15^, we found that chronic cCr treatment was effective on different aspects of CTD, including cognitive dysfunction, autistic-like features and epilepsy. The outcome of this pharmacological approach has been previously evaluated in brain-specific CrT conditional knockout mice, reporting a beneficial effect for learning and memory deficits^10^. Consistent findings across multiple outcomes would help increase confidence in the robustness and generalizability of preclinical results. The Kurosawa study, however, was mainly oriented in testing the effective ability of cCr to enter brain cells and to serve as substrate for Cr kinase, and a brain-specific rather than ubiquitous CrT knock-out model was used, representing a milder pathological endophenotype that seems to emerge quite late in development^14^. Moreover, Kurosawa and colleagues probed only cognitive aspects of CTD, used aged animals (PND365), and treated for only a short duration (8 weeks) with a single dose level (allegedly 280 mg/kg) of cCr ad libitum in drinking water^10^.

Here, we designed the study to fulfill the best-practice guidelines for assessment in preclinical studies. This included incorporation of several key design aspects to ensure clinical translatability. The use of a mouse model where there is ubiquitous CrT loss aligns better with the genetics and the early onset of CTD^11^. We verified that cCr reached the brain and the concentrations present at the end of chronic dosing duration were dose-responsive and consistent with those predicted by pharmacokinetic simulation of data previously obtained in a single oral dose biodistribution study (unpublished results, personal communication by Minh-Ha T. Do, Lumos Pharma). Moreover, we integrated robust statistical design with a priori computation of the sample size, minimization of genetic variability, random allocation of subjects to experimental groups, blinded assessment of readouts, longitudinal quantification of multiple robust and reproducible outcome measures, and dose–response analyses with placebo controls to determine the minimum efficacious dose (MED) on different aspects of the CTD pathological framework. Our results clearly indicate that the high (140 mg/kg) and the medium (46 mg/kg) doses have the stronger therapeutic effect in CrT^−/y^ mice and that the MED is potentially 46 mg/kg for cognitive defects and epileptic endophenotype, whereas 14 mg/kg may be sufficient to reduce autistic-like stereotyped behavior. This variability in the MED is likely to reflect distinct metabolic constraints of different brain processes, with the circuitry involved in the government of stereotyped movements possibly demanding less energy than those related to working and declarative memory.

Despite the progressive learning and memory deterioration reported in CrT^−/y^ mice^11^, we found that cCr administration is still able to correct the cognitive phenotype at PND180, suggesting that cumulative beneficial effects on brain energy metabolism could result in a significant regression of early aging processes set in motion by Cr deficiency. This could be in part related to the time frame when dosing was initiated in this study. While it is challenging to relate developmental age of a rodent to that of a human, a PND21 mouse is roughly equivalent to a 1–3-year-old human child by multiple measures of nervous system development^30^. Starting treatment at this age in the animal model coincides with the time point when CTD patients typically present clinically and our results indicate that early intervention may be critical. Accordingly, early treatment has been shown to be more beneficial in the other two Cr deficiency syndromes^31,32^. The finding that cCr can impact disease progression also implies that long-term pharmacological intervention with cCr does not lead to the development of tolerance and these data highlight the relevance of patient compliance with chronic treatment protocols.

Importantly, our results show that cCr treatment is able to partially correct cortical hemodynamic responses measured through IOS imaging and EEG abnormalities caused by Cr depletion^15^, demonstrating that these functional biomarkers enable tracking of disease progression and response to chronic treatment in the animal model. These data reinforce the concept that EEG and functional near-infrared spectroscopy (fNIRS, a non-invasive method for assessing hemodynamics responses in children^33,34^) might allow clinicians to optimize the follow-up of patients and to monitor the efficacy of potential therapeutic strategies on brain activity. Moreover, the study of EEG pattern could be predictive of the epileptic phenotype^15^.

We did not report any detrimental effects of cCr treatment in this study, including no cCr-related deaths, pathological alterations in brain morphology, or exacerbation of the epileptic phenotype. In fact, we observed just the opposite with cCr protecting against spontaneous and chemically-induced convulsions in CrT^−/y^ mice. The only possible side effect could be the gain of alpha power during the sleep phase observed following the treatment with the 140 mg/kg cCr dose, potentially related to sleep disturbances^35,36^. This is in contrast to a recent toxicology study where cCr has been described to increase seizure incidence and brain vacuolation in Sprague–Dawley rats^37^. This difference could be related to the higher doses used in the latter study (vacuolation was observed at 600 mg/kg/day), the different species (rats vs. mice) and/or the genotype of animals (wild type vs. animals lacking CrT function). Since cCr does not require CrT for cellular uptake, but can be transported by CrT^38^, this could potentially lead to differential build-up of cCr in wild-type compared to CrT^−/y^ animals. Taken together, these data raise the possibility that cCr could have adverse effects on brain circuitry when present in excess, but additional formal toxicology studies in different species would be needed to assess the safety window of this drug.

Overall, our findings support the therapeutic efficacy of cCr for treating CTD, laying the foundation for the design of intervention protocols for this molecule or other chemically modified, lipophilic compounds that could be readily translated to the bedside.

## Methods

### Animals

We employed male mice hemizygous for CrT exons 5–7 deletion (CrT^−/y^) on a C57Bl/6J background^39^, and their wild-type (WT; CrT^+/y^) littermates. All experiments were carried out in accordance with the European Directive of 22 September 2010 (EU/63/2010) and were authorized by the Italian Ministry of Health (authorization number 259/2016-PR and 507/2018-PR).

### Drug administration and design of efficacy study

Cyclocreatine (cCr) was dissolved in 1% low-fat chocolate milk. Dosing solutions were stored at 2–8 °C between use and fresh solutions were made weekly. CCr in 1% chocolate milk was shown to be stable for 3 weeks at room temperature, and solubility was determined to be 15 mg/mL. Animals were left untreated or presented daily with 1% low-fat chocolate milk (vehicle treatment, V) or a 1% low-fat chocolate milk-cCr cocktail (in a volume of 5 ml/kg). cCr was administered once daily at three different dose levels (140, 46 or 14 mg/kg) over the course of 24 weeks starting in mice at postnatal day (PND) 21. Longitudinal IOS imaging was conducted at PND40, PND110 and PND180. After completion of each IOS recording, mice were rested for at least four days and then each animal was subjected to serial neurobehavioral assessments of cognitive and psychomotor functions (Y maze, Morris Water Maze, rotarod and self-grooming). While Y maze, MWM and rotarod were longitudinally administered to the same animals, self-grooming was tested only at PND195 (see Fig. 1a). At the end of the dosing period (PND200), animals were monitored via EEG for 24 h and then challenged with KA. A subset of randomly selected M-CrT^−/y^ (n = 5) and L-CrT^−/y^ mice (n = 4) were not used for EEG and the brain was harvested for cCr pharmacokinetic profiling Otherwise, all animals were subjected to the same tests in the same time intervals with no differences in the experimental timeline was introduced between individuals or groups.

### Intrinsic optical signal (IOS) imaging

Surgery and imaging sessions were performed as previously described^40^. Images were visualized using a custom-made setup based on a Leica macroscope (Leica Z6 APO). Animals were fixed under the objective using a magnet mounted on an arduino-based imaging chamber. Red light illumination (630 nm) was obtained with 8 individually addressable LEDs (WS2812) secured to the objective (Leica PanApo 2.0X 10447170) by a custom 3D printed holder^41^. Visual stimuli were generated using Matlab Psychtoolbox and displayed on a screen placed 13 cm away from the eyes of the animals. Sinusoidal wave gratings were presented in the binocular portion of the visual field with spatial frequency 0.03 cyc/deg, mean luminance 20 cd/m^2^ and contrast 90%. The stimulus consisted in the abrupt contrast reversal of a grating with a temporal frequency of 4 Hz for 1 s. Frames were acquired at 30 fps with a 512 × 512 pixels resolution. The signal was averaged for at least 80 trials. Changes in reflectance (R) for each pixel were computed as the normalized difference from the average baseline (ΔR/R). A region of interest (ROI) was identified on the mean image of contralateral eye response by selecting the pixels in the lowest 30% ΔR/R of the range between the maximal and minimal pixel intensity, and mean evoked response was estimated as the average intensity within the ROI. See^40^ for further details on signal analysis.

### Behavioral tests

All behavioral tests were performed as previously reported^11^. *Y-maze*—We used a Y-shaped maze with three symmetrical plastic arms at a 120° angle (26 × 10 × 15 cm). Mice were allowed to explore the maze for 8 min. Video recordings (Noldus Ethovision XT) were employed for offline blind analysis. An arm entry was scored when all four limbs of the animal were within the arm. A triad was defined as a set of three consecutive arm entries, with each entry being into a different arm of the maze. The number of arm entries and the number of triads were recorded in order to calculate the alternation percentage. *Morris water maze*—Mice were trained for four trials per day and for a total of 7 days in a circular water tank (diameter, 120 cm; height, 40 cm), filled to a depth of 25 cm with water (21–22 °C) added with non-toxic white paint. Four alternative starting positions defined the division of the tank into four quadrants. A square clear Perspex platform (11 × 11 cm) was submerged 0.5 cm below the water surface and located at the midpoint of the target quadrant. Mice were allowed up to 60 s to climb the escape platform, while their swimming paths were recorded by the Noldus Ethovision system. On the last trial (probe trial), the platform was removed and the swimming paths were recorded over 60 s. *Rotarod*—Mice were placed on an increasingly rotating drum (speed varying from 4 to 40 rpm in 10 min). The time spent on the drum was recorded by an automated unit. Four consecutive trials with an inter-trial interval of 5 min were performed in a single day. *Self-grooming*—Each mouse was placed individually into an empty, standard mouse cage (27 length, 20 cm wide, 15 cm high). Animal behavior was recorded for 20 min. After a 10-min habituation period, each mouse was scored for 10 min for cumulative time spent grooming all body regions.

### EEG recordings

A two-channel headmount was placed on the skull of mice. EEG electrodes were stainless steel screws implanted epidurally over the frontal and the occipital areas. EEG signals were recorded using a preamplifier connected to a data acquisition system and Sirenia Software 1.7.9 (Pinnacle Technology). Signals were recorded at 400 Hz sampling frequency to evaluate baseline (spontaneous) activity for 24 h followed by treatment with i.p. kainic acid (KA) at 10 mg/kg to evoke seizure activity. Video was recorded in parallel during the entire duration of the EEG assessments. The vigilance state of the animals was classified as active wake, passive wake or sleep according to the video inspection. Signals were segmented in epochs lasting 30 s and converted into power spectra by Fast Fourier Transform: 0.5–4 (delta), 4–8 (theta), 8–12 (alpha), 12–30 (beta), and 30–45 (gamma) Hz bands were analyzed. At least 12 epochs in each light/dark cycle and wake/sleep condition were averaged. To quantify both spontaneous and KA-evoked seizure episodes we used Sirenia Seizure Pro 1.8.4. The baseline period of each animal was used as a cutoff threshold (mean line length + 8 × SD). Events in the 2–10 Hz frequency range, with a line length higher than the defined threshold, and lasting at least 10 s were identified as seizures. At the behavioral level, seizures were scored according to Racine scale^42^. Seizures of stage 1 and 2 were classified as tonic events, seizures of stage 3 were assigned to clonic events and seizures of stage 4, 5 and 6 were categorized as tonic–clonic events.

### Satellite pharmacokinetic and histology study

Satellite mice were dosed in the same manner as the efficacy study animals and used for brain pharmacokinetics (PK) and histology evaluation. CrT^−/y^ mice were either not dosed (CrT^−/y^) or treated daily with 1% low-fat chocolate milk (vehicle, V-CrT^−/y^) or 140 mg/kg cCr (H-CrT^−/y^). Of the 21 animals, 7 per group were terminated at each time point (PND44, PND114, or PND184). Twenty-four hours after the last dose, brains were harvested from the mice following whole-body perfusion with cold saline. One hemisphere was immediately snap frozen for PK study and the other hemisphere was fixed in 10% neutral-buffered formalin for histology. For PK, LC–MS/MS assays for cCr (limit of quantification 0.35 nmol/g brain) and phospho-cCr (limit of quantification 0.22 nmol/g brain) were developed and used to measure concentrations of total cCr (summation of cCr and phospho-cCr) against an internal standard (cyclocreatine-1,4,5-^13^C_3_) in the brain hemispheres of all satellite H-CrT^−/y^ animals at PND44, 114, and 184. Additionally, brain PK was measured in 4–5 randomly selected M-CrT^−/y^ and L-CrT^−/y^ animals from the efficacy study at PND195 (after whole body perfusion with saline, see “Drug administration and design of efficacy study” paragraph). For reference, brain creatine and phospho-creatine values in wild-type C57BL/6J mice were obtained in a separate study from the same laboratory that developed and ran the cCr/phospho-cCr assays. The mean total creatine value reported (12,813 nmol/g brain) is very similar to that reported by other groups in wild-type animals^10,11,43^. For histology, 5 of the 7 animals in each group were randomly selected for evaluation at PND114 and PND184. Hemispheres were trimmed into 8–10 coronal sections, embedded in paraffin, sectioned using a 5-micron block advance, and stained with Hematoxylin and Eosin. Amygdaloid body, basal nuclei/striatum, cerebellum, cerebral cortex, hippocampus, hypothalamus, medulla oblongata, meninges, midbrain, olfactory bulb, pons, thalamus ventricular system, and white matter were evaluated for each animal.

### Statistical analysis

We estimated the sample size needed by performing a power analysis using G * Power (α = 0.05, β = 0.2). The estimate of the expected difference between the experimental groups was based on the knowledge of values of the studied parameter obtained historically in our laboratory in CrT^−/y^ animals. We estimated the minimal difference that would be biologically relevant considering the impact of the possible difference on the animals’ brain function and behavior. Since we performed a longitudinal study with different measurements carried out in the same animals, we based the a priori statistical analysis on the parameter with the smallest effect size in previous experiments (Morris Water Maze). Starting from the hypothesis that cCr treatment would be effective in ameliorating the endophenotype of CrT^−/y^ mice, we established behavioral assessments as primary readouts, whereas IOS imaging, EEG recordings, PK measurements and histology were used as secondary readouts. All statistical analyses were performed using GraphPad Prism 8.0.1. The significance of factorial effects and differences among more than two groups were evaluated with ANOVA/RM ANOVA followed by post hoc Dunnett multiple comparison test. Fisher’s exact test and χ^2^ test were used to compare sampling distributions. A mixed-effects analysis was carried out for statistical evaluation of body weight. Rank transformation was exploited for data not normally distributed. The level of significance was *p* < 0.05.

## Supplementary information

Supplementary Information.

## Data Availability

The datasets generated during the current study are available from the corresponding author on reasonable request.
