# Supplementary material for: Cyclocreatine treatment ameliorates the cognitive, autistic and epileptic phenotype in a mouse model of Creatine Transporter Deficiency
Source: Sci Rep. 2020 Oct 27;10:18361. doi: 10.1038/s41598-020-75436-4 (PMC7591530; doi:10.1038/s41598-020-75436-4)
Supplement: Supplementary file 1 — Supplementary Information. [file 41598_2020_75436_MOESM1_ESM.pdf]

Cyclocreatine treatment ameliorates the cognitive, autistic and epileptic phenotype in a mouse model of Creatine Transporter Deficiency- Supplementary Information

Francesco Cacciante[1,2], Mariangela Gennaro[1], Giulia Sagona[3,4], Raffaele Mazziotti[1], Leonardo Lupori[2], Elisa Cerri[1], Elena Putignano[1], Mark Butt[5], Minh-Ha T. Do[6], John C. McKew[6], Maria Grazia Alessandrì[4], Roberta Battini[4,7], Giovanni Cioni[4,7], Tommaso Pizzorusso[1,3], Laura Baroncelli[1,4*]

[1] Institute of Neuroscience, National Research Council (CNR), I-56124, Pisa, Italy.
[2] BIO@SNS lab, Scuola Normale Superiore di Pisa, I-56125 Pisa, Italy.
[3] Department of Neuroscience, Psychology, Drug Research and Child Health NEUROFARBA, University of Florence, I-50135, Florence, Italy.
[4] Department of Developmental Neuroscience, IRCCS Stella Maris Foundation, I-56128, Pisa, Italy.
[5] Tox Path Specialists, Frederick, MD 21701, USA
[6] Lumos Pharma, Austin, TX 78756, USA.
[7] Department of Clinical and Experimental Medicine, University of Pisa, Pisa, Italy.
*To whom correspondence should be addressed: baroncelli@in.cnr.it.

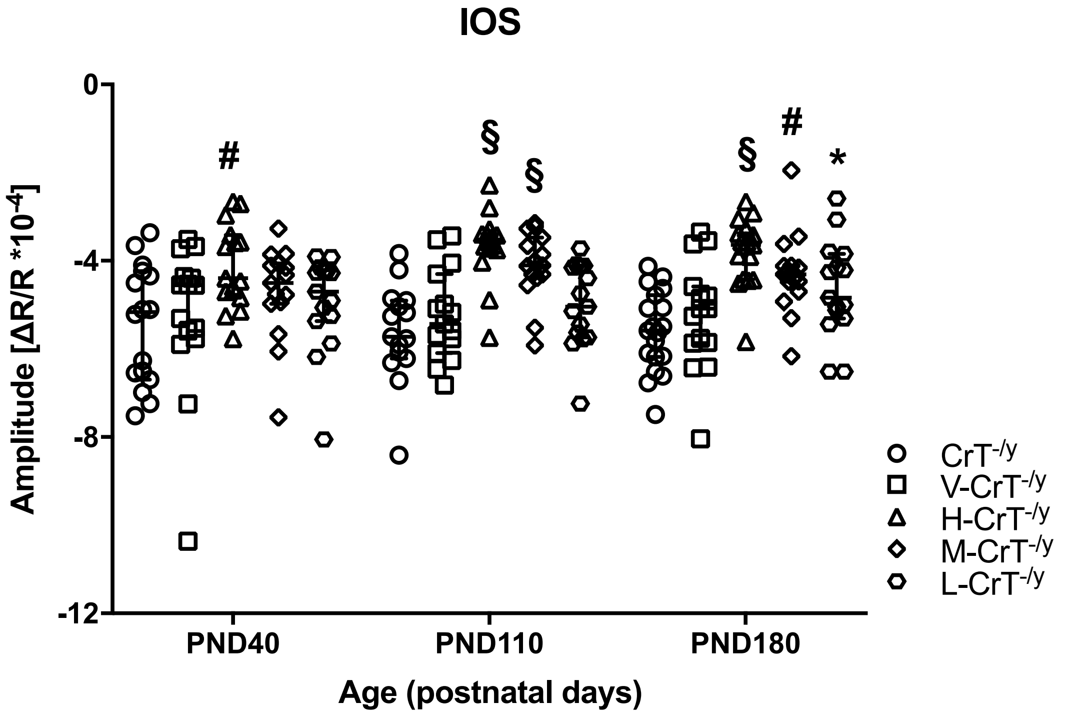

Fig. S1. cCr treatment improves hemodynamic responses in CrT-/y mice. We evaluated the therapeutic efficacy of longitudinal treatment with cCr at three different doses (high dose, H: 140 mg/kg, medium dose, M: 46 mg/kg, low dose, L: 14 mg/kg). Untreated mice ($CrT^{-/y}$) and animals administered with placebo (only chocolate milk, V-$CrT^{-/y}$) were used as controls (n = 15 for each group at each time point, except n = 17 each for H-CrT-/y and M-CrT-/y mice at PND180). The graph shows the amplitude of IOS responses following visual stimulation measured after 20 (PND40), 90 (PND110) and 160 (PND180) days of cCr delivery. Open symbols represent single data values; black lines indicate median with 95% CI. * $p < 0.05$, # $p < 0.01$, § $p < 0.001$.

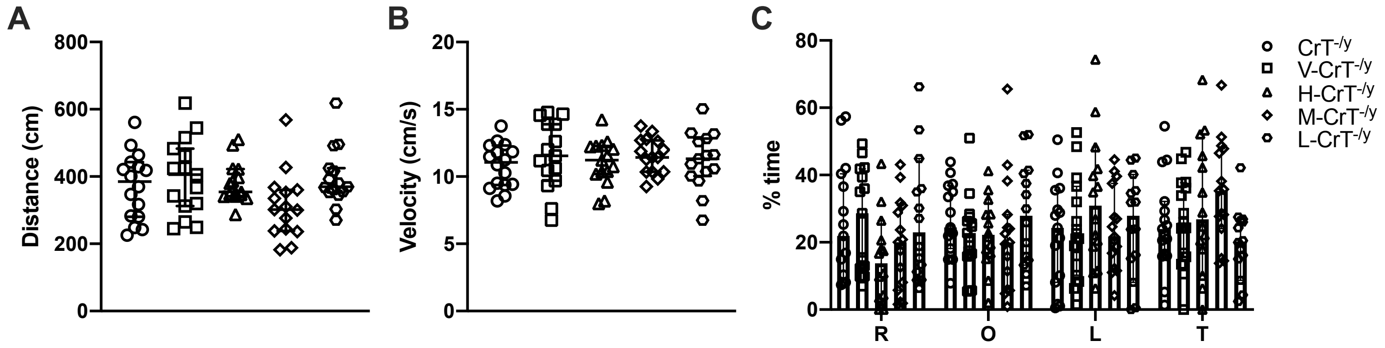

Fig. S2. Spatial learning and memory at PND116. (A) No effect of cCr was found in the average distance covered to locate the platform in the last three days of training (One-way ANOVA, $p = 0.09$) and **(B)** in swimming velocity of mice ($p = 0.469$). Open symbols represent single data values ($n = 15$ for each group); black lines indicate median with 95% CI. **(C)** The probe test shows lack of preferential exploration of the target (T) quadrant where the platform was located during the training phase for all experimental groups ($n = 15$ for each group; Two-way RM ANOVA, effect of treatment $p = 0.718$, interaction treatment x quadrant $p = 0.182$). R, right; O, opposite; L, left. Open symbols represent single data values; histograms and lines indicate median with 95% CI.

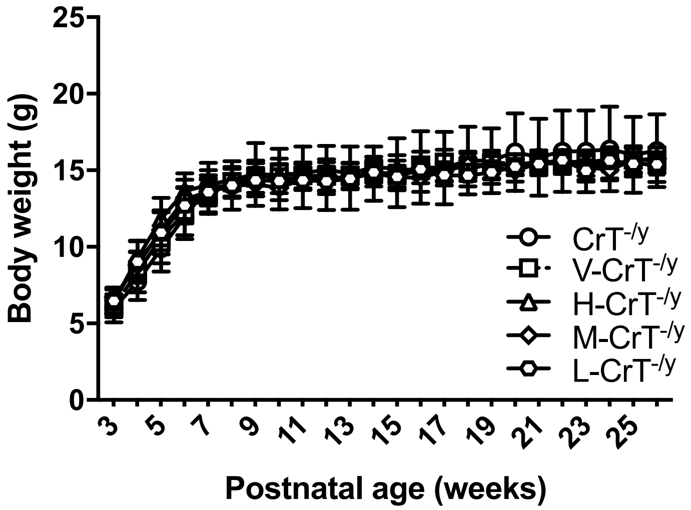

Fig. S3. No effect of cCr treatment on body weight. The weight of $CrT^{–/y}$ mice treated with the different doses of cCr ($H\text{-}CrT^{-/y}$, n = 19; $M\text{-}CrT^{-/y}$, n = 17; $L\text{-}CrT^{-/y}$, n = 16) was comparable to that of untreated (n = 18) and vehicle-treated $CrT^{-/y}$ animals (n = 16) at any age tested (mixed effects analysis, effect of treatment p = 0.989). Means with 95% CI are shown.

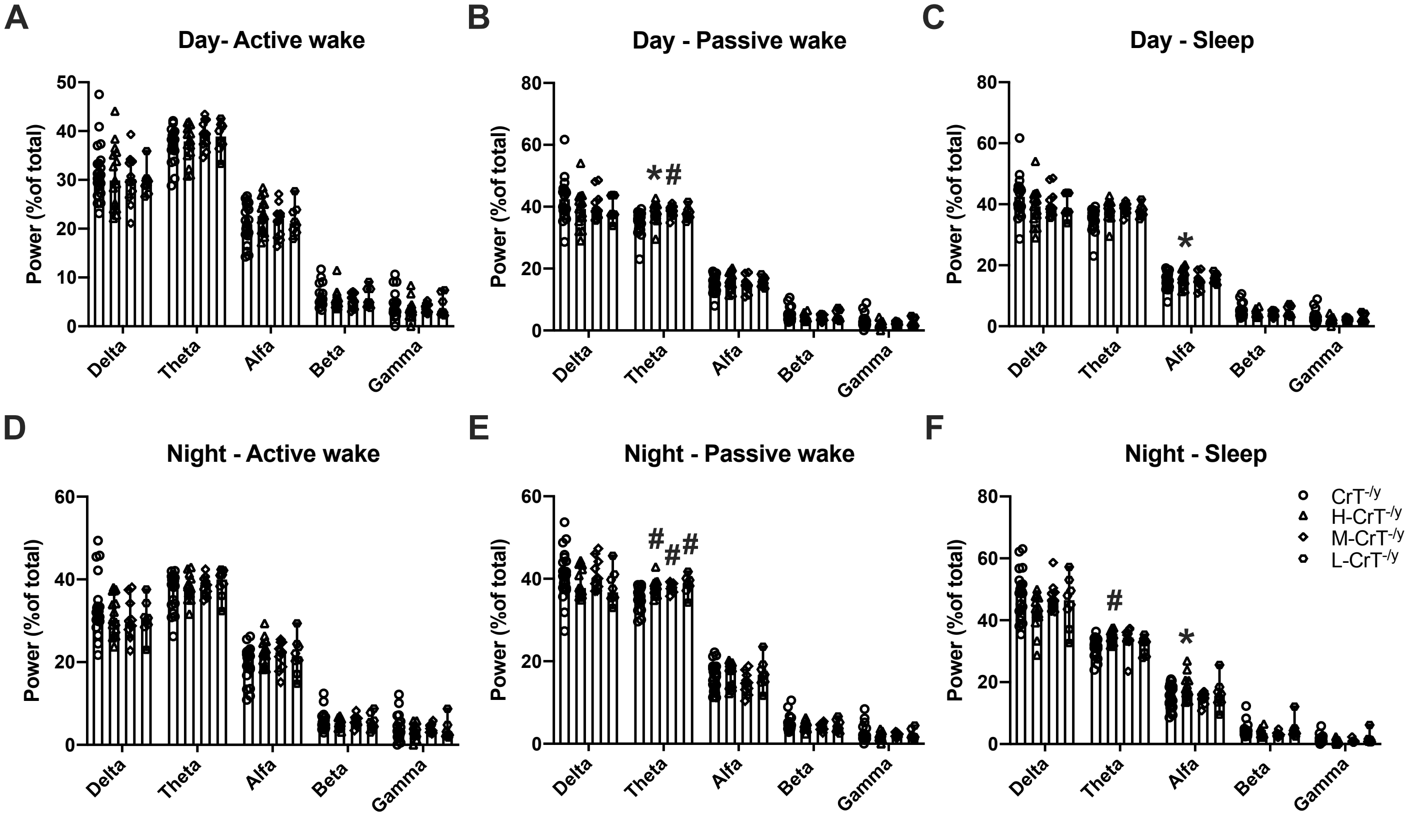

Fig. S4. Rescue of theta brain oscillations following cCr treatment. (A-F) Normalized power spectrum of cortical EEG recordings from $CrT^{-/y}$ ($n = 24$), H-$CrT^{-/y}$ ($n = 15$), M-$CrT^{-/y}$ ($n = 12$) and L-$CrT^{-/y}$ mice ($n = 9$). Untreated and V-$CrT^{-/y}$ mice were pooled together in the $CrT^{-/y}$ group. Increased power of 4-8 Hz theta frequency in H-$CrT^{-/y}$ and M-$CrT^{-/y}$ animals during the passive wakefulness (One-way ANOVA, $p < 0.01$, post-hoc Dunnett's multiple comparisons test, $p < 0.05$ for H-$CrT^{-/y}$ and $p < 0.01$ for M-$CrT^{-/y}$), and increased power of 8-12 Hz alpha frequency in H-$CrT^{-/y}$ mice ($p < 0.05$) during sleep of light phase. Increased power of 4-8 Hz theta frequency in H-$CrT^{-/y}$, M-$CrT^{-/y}$ and L-$CrT^{-/y}$ animals during the passive wakefulness ($p < 0.01$ for all comparisons), and increased power of 4-8 Hz theta ($p < 0.01$) and 8-12 Hz alpha frequency ($p < 0.05$) in H-$CrT^{-/y}$ mice during sleep of night phase. Vigilance state was classified by video inspection. * $p < 0.05$, # $p < 0.01$. Open symbols represent single data values; histograms and lines indicate median with 95% CI.

H-CrT$^{-/y}$
PND114
Mouse #33

H-CrT$^{-/y}$
PND184
Mouse #57

H-CrT$^{-/y}$
PND184
Mouse #73

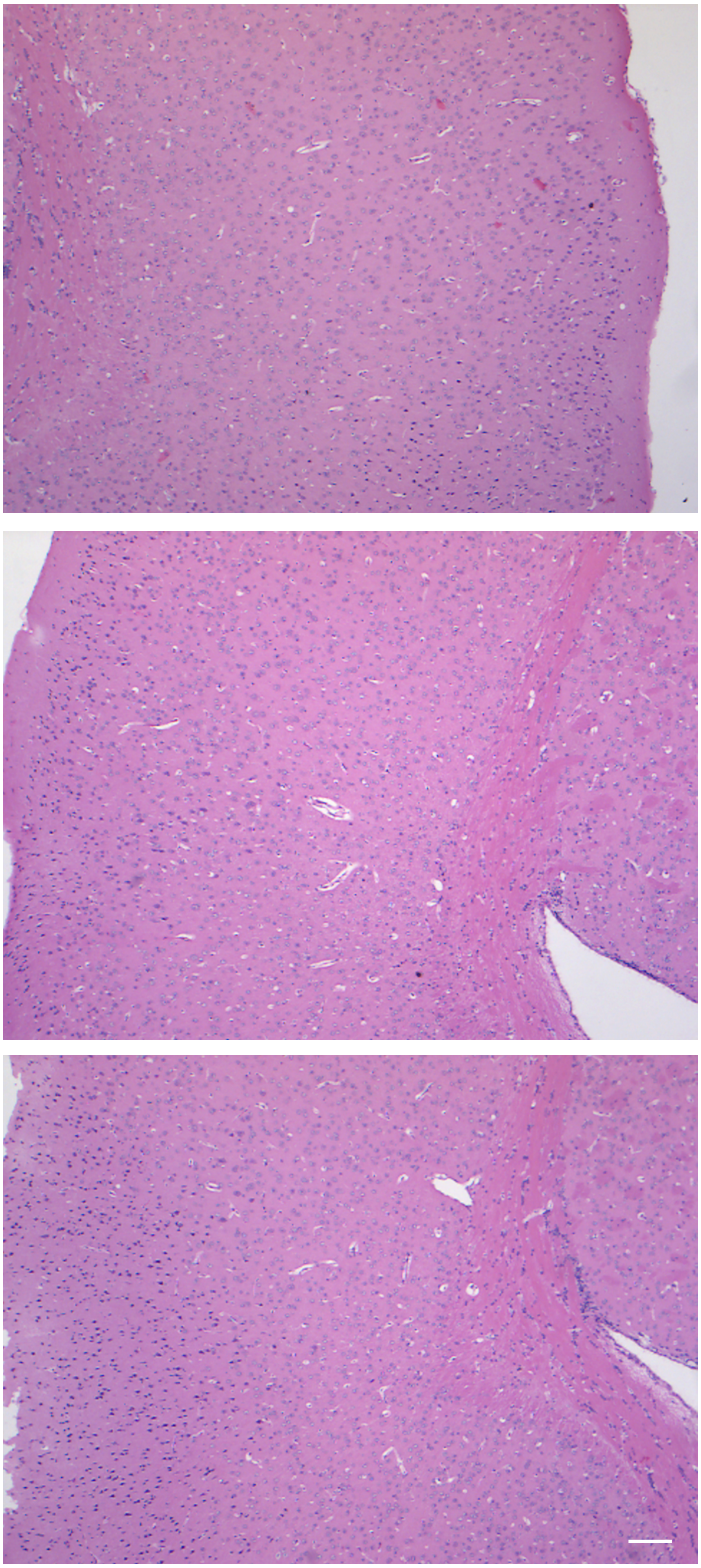

Fig. S5. Hematoxylin and Eosin histological staining in the cerebral cortex of CrT$^{-/y}$ mice dosed cCr 140 mg/kg. At the end of the dosing period, formalin-fixed brain hemispheres from randomly selected satellite animals were evaluated microscopically using Hematoxylin and Eosin staining. No pathological changes were observed following cCr administration. Representative images of the cerebral cortex from three individual mice at 4X magnification are shown. Calibration bar (bottom right): 100 um.
